# Nonlinear Mixed-Effects (NLME) Diameter Growth Models for Individual China-Fir (*Cunninghamia lanceolata*) Trees in Southeast China

**DOI:** 10.1371/journal.pone.0104012

**Published:** 2014-08-01

**Authors:** Hao Xu, Yujun Sun, Xinjie Wang, Yao Fu, Yunfei Dong, Ying Li

**Affiliations:** 1 The Key Laboratory for Silviculture and Conservation of Ministry of Education, College of Forestry, Beijing Forestry University, Beijing, PR China; 2 College of Forestry, Beijing Forestry University, Beijing, PR China; Pennsylvania State University, United States of America

## Abstract

An individual-tree diameter growth model was developed for *Cunninghamia lanceolata* in Fujian province, southeast China. Data were obtained from 72 plantation-grown China-fir trees in 24 single-species plots. Ordinary non-linear least squares regression was used to choose the best base model from among 5 theoretical growth equations; selection criteria were the smallest absolute mean residual and root mean square error and the largest adjusted coefficient of determination. To account for autocorrelation in the repeated-measures data, we developed one-level and nested two-level nonlinear mixed-effects (*NLME*) models, constructed on the selected base model; the *NLME* models incorporated random effects of the tree and plot. The best random-effects combinations for the *NLME* models were identified by Akaike's information criterion, Bayesian information criterion and −2 logarithm likelihood. Heteroscedasticity was reduced with two residual variance functions, a power function and an exponential function. The autocorrelation was addressed with three residual autocorrelation structures: a first-order autoregressive structure [AR(1)], a combination of first-order autoregressive and moving average structures [ARMA(1,1)] and a compound symmetry structure (CS). The one-level (tree) *NLME* model performed best. Independent validation data were used to test the performance of the models and to demonstrate the advantage of calibrating the *NLME* models.

## Introduction

China-fir (*Cunninghamia lanceolata* (Lamb.) Hook) is the most commonly grown afforestation species in southeast China because of its fast growth and good wood qualities. It is widely used for buildings, furniture, bridge construction and many other purposes.

Growth and yield models are commonly used for forest management planning because they can simulate stand development and production under various management alternatives [1], [2]. Individual-tree diameter growth models are a fundamental component of forest growth and yield prediction frameworks [3]–[5]. The models are based on extensive growth data obtained from diverse regions and management levels. Individual-tree diameter growth can be expressed as a function of tree size, competitive effect, stand structure and site quality [6]. A distance-independent individual-tree model structure may be flexible enough to predict diameter growth in monospecific even-aged stands and in mixed-species and multi-aged stands [7].

Regression analysis, such as ordinary non-linear least squares (*ONLS*) regression, is the most commonly used statistical method in forest modeling [8]. Individual-tree diameter growth models have been fitted to growth increment data collected repeatedly over time on the same tree [9]. The hierarchical nature of the data results in spatial and temporal correlation among observations made in the same sampling unit (i.e., plot and tree) [10]. However, the stochastic structure is often ignored and independence of observations is assumed [11]–[15]. Furthermore, the data are autocorrelated and cannot be considered independent samples of the basic tree population [10]. The *ONLS* regression assumption of independent residuals is therefore violated, biasing the estimates of the standard error of the parameter estimates [16]. Many recent efforts to develop diameter growth models have used nonlinear mixed-effects (*NLME*) models [5], [17].

*NLME* models include both fixed effects, which are parameters associated with an entire population or with certain repeatable levels of experimental factors, and random effects, which are associated with individual experimental units drawn at random from a population [18]. Random effects account for spatial and temporal correlation by defining the covariance structure of the model's random components and by using this structure during parameter estimation. *NLME* models provide an efficient statistical method for explicitly modeling hierarchical stochastic structure. Growth models can be calibrated by predicting random components from tree- or plot-level covariates when a new subject is available and is not used in the fitting of the model by using the empirical best linear unbiased predictors (EBLUPs) [3], [4], [12], [19].

Statistical models in which both fixed and random effects enter nonlinearly are increasingly common in the biosciences [20]. The models are relevant to many disciplines, including forestry, agriculture, ecology, biology, biomedicine and pharmacokinetics [21]. They are used to analyze data with complex structures, including grouped data, longitudinal data, repeated measures data and multivariate multilevel data [22]. One of the most common applications is for analysis of nonlinear growth data [23]; these are data measured repeatedly over time on the same tree (multiple observations obtained from the same sampling unit or subject in sequence over time) and are also known as longitudinal data [12], [24].

The main purpose of this study was to develop an individual-tree diameter growth model for *C. lanceolata* (Lamb.) Hook growing in Fujian province, southeast China. The data were derived from 144 increment cores from 72 trees in 24 sample plots. One-level and nested two-level nonlinear mixed modeling approaches that included both fixed and random components were applied to the hierarchical structure of the data. This diminished the level of variance among the sampling units, which were included as covariates. In developing the diameter growth models, we considered nested two-level models and a single-level model. The first level is the plot and the second level is the tree, nested within the plot. Our preliminary analysis showed that the *NLME* models with random effects effectively removed the heteroscedasticity and autocorrelations in the repeated-measure data and therefore could be important tools for sustainable management of China-fir species within the study area. The predictive ability of the developed model and the applicability of the *NLME* model were demonstrated using separate validation data.

## Materials and Methods

### Data

**Figure 1:**
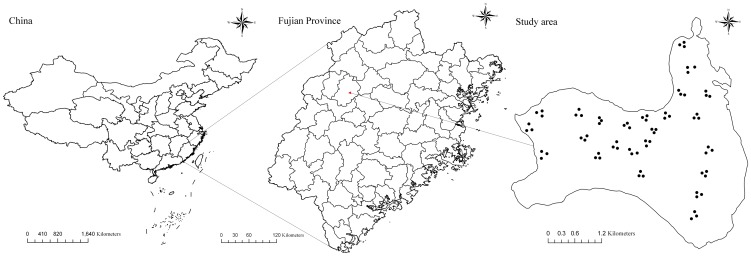
Seventy two trees in twenty four sample plots on Jiangle state-owned forest farm in southeast China.

The data were obtained from 24 single-species plots of plantation-grown China-fir on the Jiangle state-owned forest farm in southeast China (Figure 1). One-hundred and forty-four increment cores were collected from 72 trees; 15 cores missed the pith and were excluded from analysis. The increment cores were extracted from three mean trees in each plot; the mean trees were trees with a diameter at breast height (dbh; 1.3 m above ground) approximately equal to the plot mean dbh. Two cores were collected perpendicular to each other from each tree at breast height. The sample plots were square and varied in size from 400 to 600 m^2^. All standing live trees (height >1.3 m) on the plots were measured for dbh (outside bark) and tree height. Three to five dominant trees on each plot were chosen to calculate plot dominant height. Using the Lintab tree-ring measurement system (Rinntech Company in Germany), the width of each annual growth ring (radial increment data) was measured; trees were assumed to be round, so the diameters were calculated as twice the radius. Growth data from the seed orchard at the forest farm indicate that China-fir requires two years to attain a height of 1.3 m. Diameter data were therefore assigned an initial age of 3 years. Independent data were used for model validation. The data were randomly divided into two groups; 75% of the points were used for model fitting, and 25% were used for model validation. The fitting data and the validation data included 54 trees from 23 plots and 18 trees from 13 plots, respectively. Summary statistics for both fitting and validation data are shown in Table 1.

**Table 1:** Summary statistics for both fitting and validation data.

Data	Variable	Min	Max	Mean	sd	Data	Variable	Min	Max	Mean	sd
Fitting data	QMD (cm)	10.2	23.2	17.4	3.69	Validation data	QMD (cm)	10.2	25.2	17.9	5.19
	MT(m)	5.0	21.3	16.0	3.13		MT (m)	5.0	21.3	15.9	3.88
	SD (tree ha^−1^)	800	4400	1905	849.68		SD (tree ha^−1^)	717	4400	1973	1168.08
	DD (cm)	10.1	25.7	21.8	3.28		DD (cm)	10.1	24.8	21.5	3.54
	DH (m)	7.3	30.3	21.6	4.12		DH (m)	7.3	26.3	20.4	3.91
	*H* (m)	7.1	27.7	18.3	4.82		*H* (m)	7.0	22.7	16.8	4.16
	BA (m^2^ ha^−1^)	15.67	68.00	37.24	14.12		BA (m^2^ ha^−1^)	15.67	59.43	37.72	13.19
	SA (m)	176	320	226	32.61		SA (m)	176	320	226	38.82
	SS (°)	15	41	29	6.32		SS (°)	23	41	32	3.50
	SI (m at 20 years)	12	24	20	2.83		SI (m at 20 years)	12	24	18	3.50
	SAG (yr)	5	37	24	6.98		SAG (yr)	6	38	26	8.79

QMD, plot quadratic mean diameter; MT, mean tree height of forest stand; SD, stand density; DD, plot dominant diameter; DH, plot dominant height; *H*, sample tree height; BA, basal area; SA, stand altitude; SS, stand slop; SI, site index; SAG, stand age; sd, standard deviation.

### Methods

#### Nested two-level NLME model

The model data were derived from the measured annual increment of the sampled trees. The nested sampling structure created a high degree of correlation among observations taken from the same tree and plot. The mixed-effects modeling approach is a common means of addressing the correlation structure in the data [13], [23]. A general expression for a *NLME* model can be defined as [22], [25].
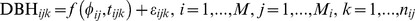
(1a)where *M* is the number of plots, *M_i_* is the number of trees within the *i*th plot, and *n_ij_* is the number of observations (increments). DBH*_ijk_* is the dbh (cm) at the *k*th age of the *j*th tree taken from the *i*th plot, *t_ijk_* is the age, *φ_ij_* is the parameter vector *r*×1 (where *r* is the number of parameters in the model), *f* is a nonlinear function of the predictor variables and the parameter vector, and *ε_ijk_* is the within-group error including the within-group variance and correlation [26]; the error is assumed normally distributed with a mean of zero and a positive-definite variance-covariance structure *R_ij_*, generally expressed as a function of the parameter vector *δ* [27].
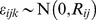
(1b)


Moreover, *φ_ij_* can be expressed as:
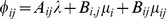
(2a)

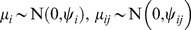
(2b)where *λ* is the *p*×1 vector of fixed population parameters (where *p* is the number of fixed parameters in the model). 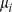 and 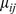 are the 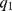
×1 and 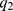
×1 vectors of random effects associated with the first and second levels, respectively (where 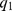 and 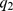 are the numbers of random parameters of two-level in the model), which are assumed to be normal (or Gaussian) with a mean of 0 and have the variance-covariance matrices *ψ_i_* and *ψ_ij_*; these are the 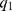
×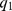 and 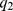
×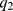 variance-covariance matrices associated with the first and second level random effects, respectively. 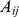, 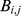
and 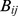 are the design matrices *r*×*p*, *r*×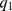 and *r*×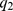 for the fixed and random effects specific to each sampling unit.

#### Individual-tree diameter growth equation

Five theoretical nonlinear growth equations, the Richards, Weibull, Korf, Logistic and Schumacher equations, were selected as candidates for modeling diameter growth. These equations are widely used for the simulation of individual-tree growth, particularly the Richards and Korf equations. Mathematical expressions of the equations are shown in Table 2.

**Table 2:** Mathematical expressions of the five equations.

Equation	Expression	Inflection point		Parameters
		Abscissa	Ordinate	
Richards	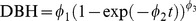	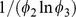	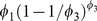	*φ*_1_, *φ*_2_>0
Weibull	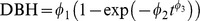	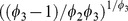	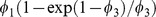	*φ*_1_, *φ*_2_, *φ*_3_>0
Korf	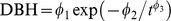	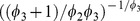	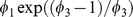	*φ*_1_, *φ*_2_, *φ*_3_>0
Logistic	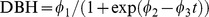	*φ*_2_/*φ*_3_	*φ*_1_/2	*φ*_1_, *φ*_3_>0
Schumacher	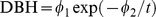	*φ*_2_/2	*φ*_1_ *e* ^−2^	*φ*_1_, *φ*_2_>0

*φ*_1_, *φ*_2_ and *φ*_3_ are the formal parameters.

The five above-mentioned equations are all S-shaped growth equations with inflection points and asymptotes. A characteristic of the Richards, Weibull and Korf equations is that the coordinates of the inflection points are variable multiples of asymptotic values; in contrast, the equivalent values of the logistic and Schumacher equations are fixed multiples [28]. The five equations were initially fit by *ONLS* regression using the R *nls* function without random parameters. Different initial values for the parameters were tried to ensure that a global minimum was achieved. The best performing function was selected as the base model by applying three statistical criteria; absolute mean residual (*AMR*), root mean square error (*RMSE*), and adjusted coefficient of determination (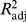) [29]. The function with the smallest *AMR* and *RMSE* and the largest 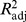 provides the best fit. The adjusted coefficient of determination is used similarly as an unbiased estimator in both multiple regression and canonical redundancy analysis. The formulas of the fit statistics are:
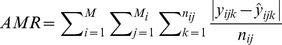
(3)

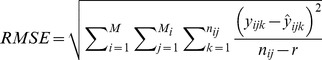
(4)

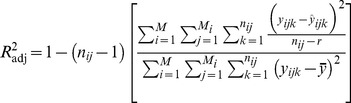
(5)where 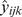 is the predicted increment at the *k*th age within the *j*th tree within the *i*th plot. 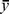 is the average of observations.

#### Mixed parameter evaluation

A crucial issue in fitting mixed-effects models is deciding which parameters should be considered random effects and which can be treated as fixed effects. A common approach is to start with random effects for all parameters and then to examine the fitted object to decide which, if any, of the random effects can be eliminated from the model [18]. Different combinations of model parameters were therefore tested to ascertain their contribution to predictions of diameter growth; the best model was selected by Akaike's information criterion (AIC) [30], Bayesian information criterion (BIC) [31] and −2 log-likelihood (−2 LL) [32]. The best model gave the smallest AIC, BIC and −2 LL. The appropriate variance function and autoregressive structure for the *NLME* models were determined by the likelihood ratio test (LRT) [18], [33]. All *NLME* models presented in this paper were calibrated using the *nlme* function in the R statistical environment [34].

#### Determining the variance-covariance structure

The variance-covariance matrices *ψ_i_* and *ψ_ij_* are positive-definite and symmetric, which is to say that all their eigenvalues must be strictly positive [18]. A hypothetical 2×2 variance-covariance matrix is shown as follows [24], [35]:
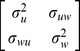
where 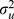 and 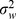 are the variance for the random effects *u* and *w*, respectively, and 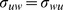 is the covariance between random effects *u* and *w*.

#### Determining the structure of *R_ij_*

The matrix *R_ij_* is allowed to depend on both random and fixed effects, as well as on a set of common but unknown parameters. The matrix accounts for within-plot heteroscedasticity and autocorrelation [24], [26], [27] by including both correlation effects and weighting factors. The matrix is expressed as [24], [36]:
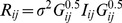
(6)where for a tree *j* in plot *i*, with *n_ij_* increment, *R_ij_* is the *n_ij_*×*n_ij_* within-tree variance-covariance matrix that defines within-group variability, *G_ij_* is an *n_ij_*×*n_ij_* diagonal matrix of within-tree error variance (heteroscedasticity), *I_ij_* is an *n_ij_*×*n_ij_* matrix of within-tree autocorrelation of the errors, and *σ* ^2^ is a scaling factor for the error dispersion [13].

In individual-tree diameter growth models, the variance is often found dependent on the means, and the variance will generally increase with increasing mean tree diameter. To remove this effect, we modeled the variance as an exponential function or power function which was used for *G_ij_* matrix [18]. And for the exponential function and power function, the diagonal elements of *G_ij_* are 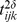 and 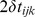, respectively, and the off-diagonal elements are all 0.
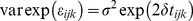
(7)

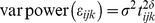
(8)


Autocorrelation structures were used for *I_ij_* matrix to address the within-tree autocorrelations of the errors observed in the data [37], [38]. A method was selected from among three commonly used approaches: first-order autoregressive structure [AR(1)], a combination of first-order autoregressive and moving average structures [ARMA(1,1)], and the compound symmetry structure (CS) [18].
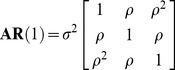
(9)

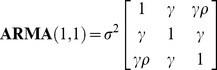
(10)

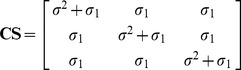
(11)where *ρ* is the autoregressive parameter, *γ* is a moving average component, *σ* ^2^ is the residual variance, and *σ*_1_ is the residual covariance [37]–[40].

#### Parameter estimation

The parameters in the equations were estimated by maximum likelihood (ML) using the Lindstrom and Bates (LB) algorithm implemented in the R *nlme* function [18], [22]. The LB algorithm and *nlme* function are detailed in several articles (see, for example, [18], [22]).

Predicting the random effects parameters is more problematic during model application and prediction than during the fitting process. In this case, they were estimated by the EBLUPs [25], using the increment data.
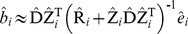
(12)where 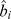 is the estimated random effects vector of EBLUPs, 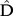 is the *q*×*q* estimated variance-covariance matrix (*q* is number of random-effects parameters) for the random effects, 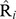 is the estimated variance-covariance matrix for the error term, 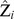 is the estimated partial derivatives matrix with respect to the random effects parameters for the new observation, and 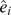 is the residual vector, whose dimension is the number of observations, and whose components are given by the difference between the observed diameter growth value for each tree, and the value predicted by the model including only fixed effects.

The standwise calibration was used to evaluate the accuracy of the calibration [12]. This type of calibration involves using the random plot components predicted from the increments of a small sample of trees per plot to predict the increment of the trees within the plot not used in the calibration. In this case, the calibration was made with 1, 2 and 3 trees per plot. The random parameters of new observations could be predicted with Equation 12.

## Results

### Function selection

The R *nls* function was used to evaluate the parameter estimates and model fit statistics of the five equations (Table 2); the results are listed in Table 3. The Korf equation had slightly better predictive ability than the others. Therefore, the Korf equation was selected as the basic nonlinear model for estimating diameter growth. The final base model is given by:
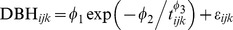
(13)


**Table 3:** Performance criteria for individual-tree diameter growth equations.

Equations	Fitting data			Validation data		
	*AMR*	*RMSE*	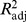	*AMR*	*RMSE*	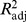
Richards	2.2169	3.3224	0.7729	3.7228	4.7236	0.7448
Weilbull	2.2724	3.3503	0.7690	3.7646	4.7196	0.7452
Korf	2.1286	3.2710	0.7855	3.6101	4.5369	0.7641
Logistic	2.3789	3.4523	0.7548	4.0064	5.0181	0.7120
Schumacher	2.1390	3.2808	0.7785	3.5979	4.6037	0.7576

### *NLME* model construction

The approach used to construct the *NLME* models was to fit the models with nested effects of plot and tree for Equation 13 and then to successively remove the random effects. The results are listed in Table 4. Four of the *NLME* models reached convergence with nested effects of plot and tree; the fifth and sixth models converged when the one-level models included the random effects of plot and tree, respectively.

**Table 4:** Evaluation indices of each *NLME* model.

Effects	Mixed parameters	AIC	BIC	−2LL
Nested effects of plots and trees	*φ*_1_	2992.3380	3022.0250	2980.3380
	*φ*_2_	not converge		
	*φ*_3_	3649.1130	3678.8010	3637.1140
	*φ*_1_, *φ*_2_	2164.7940	2214.2730	2144.7940
	*φ*_1_, *φ*_3_	2080.9480	2130.4270	2060.9480
	*φ*_2_, *φ*_3_	not converge		
	*φ*_1_, *φ*_2_, *φ*_3_	not converge		
Plots effects	*φ*_1_	5151.7190	5176.4590	5141.7200
	*φ*_2_	5744.9100	5769.6500	5734.9100
	*φ*_3_	5209.6700	5234.4100	5199.6700
	*φ*_1_, *φ*_2_	5146.6480	5181.2840	5132.6480
	*φ*_1_, *φ*_3_	5145.6490	5180.2850	5131.6500
	*φ*_2_, *φ*_3_	5168.1260	5202.7620	5154.1260
	*φ*_1_, *φ*_2_, *φ*_3_	not converge		
Trees effects	*φ*_1_	2995.6300	3020.3690	2985.6300
	*φ*_2_	4145.6660	4170.4060	4135.6660
	*φ*_3_	3651.0750	3675.8150	3641.0760
	*φ*_1_, *φ*_2_	2167.8120	2202.4480	2153.8120
	*φ*_1_, *φ*_3_	2083.0270	2117.6630	2069.0280
	*φ*_2_, *φ*_3_	2102.0420	2151.5210	2082.0416
	*φ*_1_, *φ*_2_, *φ*_3_	not converge		

LRT, AIC, BIC and −2 LL fit statistics were compared among different combinations of random effects parameters (Table 4). The models represented by Equations 14–16, incorporating the nested effects of plot and tree, plot effects and tree effects on 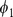 and 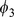, yielded the smallest AIC, BIC and −2 LL.
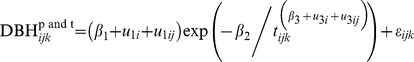
(14)

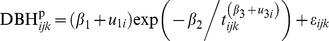
(15)

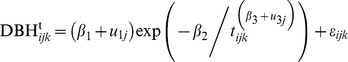
(16)where 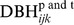 and 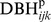, 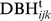 are the diameters at breast height for the three effects; 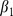, 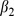 and 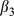 are fixed-effects parameters; 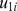 and 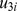 are random-effects parameters generated by plot on 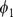 and 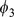, respectively; 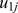 and 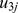 are random-effects parameters generated by tree on 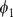 and 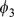, respectively; and 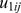 and 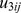 are random-effects parameters generated by interaction of plot and tree on 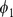 and 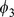, respectively.

### *NLME* models with heteroscedasticity and autocorrelation

We used the power function or the exponential function as the variance functions and the AR(1), ARMA(1,1) or CS as the autocorrelation structures to fit diameter growth models incorporating different random effects. The results of the models provided the best fit are shown in Table 5. The selected models had the smallest AIC, BIC and −2 LL. Thus, the final models of plot effects, tree effects and the two nested effects are, respectively:
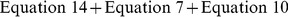
(17)

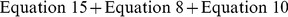
(18)

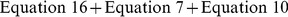
(19)


**Table 5:** Performance criteria for the best *NLME* models.

Effects	Mixed parameters	AIC	BIC	−2LL	LRT	*p* Value
Plots effects	*φ*_1_	5151.72	5176.46	5141.72		
	*φ*_1_, *φ*_3_	5145.65	5180.29	5131.65	10.07	0.0065
	*φ*_1_, *φ*_3_ with exponential function and ARMA(1,1)	2042.40	2091.88	2022.40	3109.24	<0.0001
Trees effects	*φ*_1_	2995.63	3020.37	2985.63		
	*φ*_1_, *φ*_3_	2083.03	2117.66	2069.03	916.60	<0.0001
	*φ*_1_, *φ*_3_ with exponential function and ARMA(1,1)	1113.96	1163.44	1093.96	975.07	<0.0001
Nested effects of plots and trees	*φ*_1_	2992.34	3022.03	2980.34		
	*φ*_1_, *φ*_3_	2080.95	2130.43	2060.95	919.39	<0.0001
	*φ*_1_, *φ*_3_ with exponential function and ARMA(1,1)	1112.75	1177.07	1086.75	974.20	<0.0001

### Parameter estimates

#### Nested effects of plot and tree

The nested two-level *NLME* diameter growth model is:
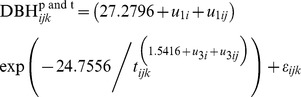
(20a)where
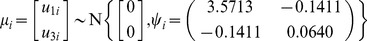
(20b)

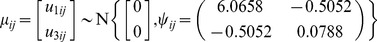
(20c)

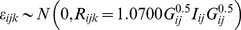
(20d)

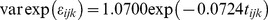
(20e)

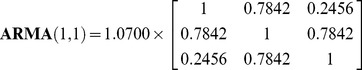
(20f)


#### Plot effects

The *NLME* diameter growth model incorporating the effect of plot is:
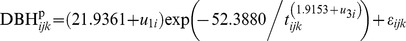
(21a)where
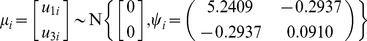
(21b)

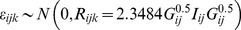
(21c)

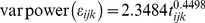
(21d)

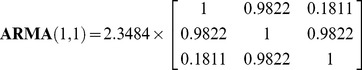
(21e)


#### Tree effects

The *NLME* diameter growth model incorporating the effect of tree is:
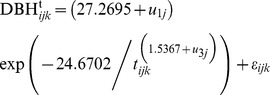
(22a)where
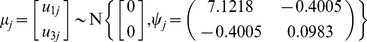
(22b)

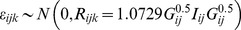
(22c)

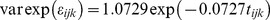
(22d)

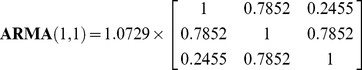
(22e)


### Model prediction

The predictive ability of Equation 13 was evaluated using predict procedures and Equations 3–5 on both fitting and validation data. The performance of the *NLME* models, with and without modeling the error structure, was evaluated using cross-validation procedures for both fitting and validation data; the random effects were predicted with the EBLUPs (Equation 12), using the measurement data.

**Figure 2:**
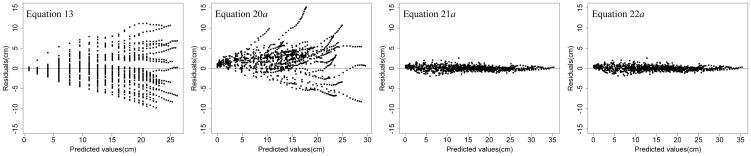
Residual error map of diameter growth of each model.

**Figure 3:**
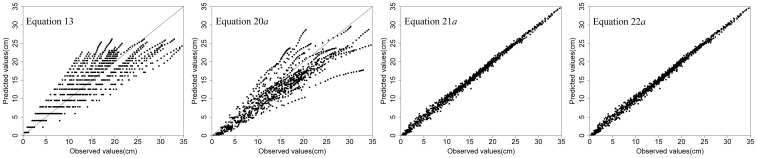
Scatter plot of fitted values against observed values of diameter growth of each model.

Table 6 lists the three fit statistics for Equation 13 and Equations 20*a*–22*a* with and without random effects. Equation 20*a* was the best predictor, with increases in 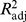 and decreases in *AMR* and *RMSE* for both fitting and validation data, but it was more complex than the others and incurred significant computing cost. In Figure 2, the residuals of Equations 13 and 20*a*–22*a* are plotted against the fitted values; the fitted values are plotted against the observed values in Figure 3. Based on the above analysis, we can conclude that, although Equation 20*a* is the strongest predictor, it is more complex than Equation 22*a* and the difference between them is small. Compared with Equation 13, Equation 22*a* had a higher 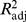, 0.9956 compared to 0.7758, and a lower *RMSE*, 0.5344 compared to 3.2810. Therefore, the *NLME* model incorporating the random effect of trees was the best model for predicting diameter growth of individual China-fir trees in the single-species plantations of the study area.

**Table 6:** Performance criteria of each model.

Equation	Effect	Fitting data			Validation data		
		*AMR*	*RMSE*	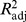	*AMR*	*RMSE*	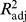
Equation 13		2.1286	3.2810	0.7785	3.6101	4.6369	0.7541
Equation 20a	Fixed effects	3.1584	2.4843	0.7251	4.0866	5.7676	0.6217
	Mixed effects	2.1722	3.6806	0.7929	2.9457	4.6317	0.7560
Equation 21a	Fixed effects	2.9172	2.0876	0.8059	3.8138	4.9368	0.7228
	Mixed effects	0.4027	0.5350	0.9956	0.6816	2.0699	0.9513
Equation 22*a*	Fixed effects	2.9170	2.0875	0.8059	3.8204	4.9357	0.7229
	Mixed effects	0.4025	0.5344	0.9957	0.6804	1.8842	0.9596

## Discussion

Of the 5 theoretical growth equations tested, the Korf equation best fit the individual-tree diameter growth data of China-fir when evaluated on the basis of *AMR*, *RMSE* and 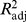. The Korf equation is widely used for forest growth and yield simulation models [41]–[43]. *ONLS* regression is commonly used to build forest growth models, but its value is limited because tree data typically violate the assumption of independent and identically distributed errors [13], [44], [45]. *NLME* models are a useful tool for analyzing repeated measures data and spatially correlated data [18], [33]. A model can be constructed with a unique variance-covariance structure that eliminates the influence of the random effects (plot and tree effects in this study). The two primary challenges in fitting *NLME* models are determining the mixed parameters and calculating the random effects [9], [18], [24], [33], [46]. An additional source of inherent correlation would be the effect of year, where observations coming from the same year would be highly correlated; tree-ring width is largely related with yearly climate variables [47]. However, year effects were not analyzed in this study. Incorporating annual climate factors into the *NLME* models may be an appropriate area for future research.

The Korf equation has been widely used as the base *NLME* model for forest growth and yield prediction. For example, Cheng and Gordon [48] successfully used the Korf equation with *NLME* models to fit loblolly pine (*Pinus taeda* L.) diameter-age relationships; the one-level (tree) individual-tree *NLME* model, based on the Korf equation, with random effects parameters 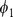 and 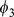 had the best fit. Parameters 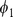, 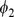 and 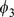 are the asymptotic values, the values associated with the growth rate of the tree and the values associated with the curve shape (inflection point) of the Korf equation, respectively. Therefore, the random effects (tree) mainly influence the maximum value and the inflection point, with evidence that the growth rate of the tree affects the model fit.

Sometimes, no prior information is available from which the random parameters can be predicted. In this case, the mixed-effects model with the random parameters set to 0 is not the same as the population average model and will give biased predictions. Instead, the population average model, fit without random effects, should be used.

## Conclusions

Five theoretical growth equations were evaluated for estimating the diameter growth of China-fir trees grown in monospecific plantations in Fujian province, southeast China. The equations can be evaluated for both biological and statistical meaning. All 5 equations and the Korf equation in particular were commonly and successfully used to model individual-tree diameter growth. One-level (plot or tree) and nested two-level (tree nested within plot) *NLME* models based on the Korf equation, with variance functions and correlation structures, were used to estimate diameter growth of individual trees; this approach was necessitated by the hierarchical structure of the experimental design and the autocorrelated tree-ring data. The results showed that the one-level (tree) *NLME* model (Equation 22*a*) with random effects was better than the others (Equations 13, 20*a* and 21*a*) (Table 5, Figure 2 and 3). Therefore, we recommend using nonlinear mixed-effects models to estimate individual-tree diameter growth.
